# Seated Sedation-Analgesia With Preserved Spontaneous Ventilation to Facilitate Intubation After Transition to the Supine Position: A Case Report

**DOI:** 10.7759/cureus.106349

**Published:** 2026-04-02

**Authors:** Ryo Tagawa, Tomoharu Shakuo, Yutaro Yamazaki, Nagisa Masunaga, Kenji Shida

**Affiliations:** 1 Department of Anesthesiology, Showa Medical University Northern Yokohama Hospital, Yokohama, JPN

**Keywords:** airway management, obesity, remimazolam, semi-sitting position, tracheal intubation

## Abstract

Induction of general anesthesia in the sitting position may be associated with hemodynamic instability resulting from decreased venous return, as well as challenges in airway management due to limited access to the airway. However, in patients who cannot tolerate the supine position because of post-traumatic pain or other conditions, induction in the sitting position may be unavoidable. In this report, we describe a case in which sedation was initiated in the sitting position, spontaneous respiration was preserved during the transition to the supine position, and tracheal intubation was subsequently performed, resulting in safe anesthetic management. The patient was a woman in her 40s with obesity who was scheduled to undergo robot-assisted surgery for a uterine malignancy associated with atypical endometrial hyperplasia. She had a history of a lumbar vertebral compression fracture secondary to a road traffic accident and was unable to tolerate the supine position while awake. After arrival in the operating room, remimazolam infusion was initiated at 6 mg·kg⁻¹·hour⁻¹ under supplemental oxygen. The patient was then moved to the supine position while maintaining spontaneous respiration. Following the administration of rocuronium and remifentanil, tracheal intubation was performed using a video laryngoscope. No complications, including decreased peripheral oxygen saturation, were observed during induction. The patient’s postoperative course was uneventful without hypoxemia after extubation, and she was discharged on postoperative day 4. In patients with obesity who cannot tolerate the supine position due to severe pain, an induction strategy using remimazolam that preserves spontaneous respiration in the semi-sitting position while transitioning to the supine position may be a useful option. When performing induction in the sitting position, developing an anesthetic plan centered on agents with available antagonists may further enhance patient safety.

## Introduction

Induction of general anesthesia in the sitting position may result in hemodynamic alterations, including decreased venous return due to gravitational effects and an increased risk of cerebral ischemia secondary to reduced cerebral perfusion pressure [1]. Furthermore, airway access and maneuverability may be restricted by patient positioning, which can make airway management procedures, including tracheal intubation, more challenging [2]. Conversely, in patients who cannot tolerate the supine position because of trauma or severe pain, induction in the sitting position may be unavoidable [2,3]. In such cases, a carefully planned perioperative strategy is required. In this report, we describe a patient with severe pain secondary to a lumbar compression fracture sustained in a motor vehicle accident, which precluded tolerance of the supine position while awake. The patient was scheduled to undergo robot-assisted surgery for a uterine malignancy associated with atypical endometrial hyperplasia. Sedation was initially performed in the sitting position, after which the patient was moved to the supine position while preserving spontaneous respiration, followed by tracheal intubation. Anesthesia was safely administered without perioperative complications. This case report was prepared in accordance with the CARE guidelines for case reports established by the EQUATOR (Enhancing the QUAlity and Transparency Of health Research) Network. Written informed consent for publication was obtained from the patient.

## Case presentation

The patient was a woman in her 40s with a height of 155 cm and a weight of 89 kg, corresponding to a body mass index of 37.0 kg·m⁻². She was scheduled to undergo robot-assisted surgery for a uterine malignancy associated with atypical endometrial hyperplasia. Her medical history included a lumbar compression fracture sustained at the age of 25 years in a motor vehicle accident. Because of chronic low back pain, she was unable to tolerate the supine position while awake and typically slept in the lateral or sitting position. She had a medical history of hypertension and schizophrenia. Preoperative evaluation at the anesthesia clinic revealed no loose teeth, limitation in mouth opening, or restriction of cervical extension. Mouth opening was approximately three fingerbreadths, the Mallampati classification was class II, and the cervical circumference was 55 cm. She had no prior diagnosis of obstructive sleep apnea and did not use home continuous positive airway pressure, bilevel positive airway pressure, or home oxygen therapy. Based on the documented preoperative findings, the STOP-BANG score was 3, with 1 point each for hypertension, body mass index >35 kg·m⁻², and neck circumference >40 cm. Her baseline peripheral oxygen saturation on room air was 97%.

The patient had no known drug or food allergies. She had a 10-year smoking history of 20 cigarettes per day, but had since quit. Her regular medications included zolpidem (10 mg/day), mirogabalin besylate (10 mg/day), loxoprofen (120 mg/day), etizolam (0.5 mg as needed for agitation), and aripiprazole (9 mg/day). Laboratory investigations, including routine blood tests, biochemical analyses, chest radiography, electrocardiography, and spirometry, revealed no significant abnormalities. Notably, six months before the current surgery, she had undergone endometrial curettage under sedation and required emergency tracheal intubation because of oxygen desaturation.

**Figure 1:**
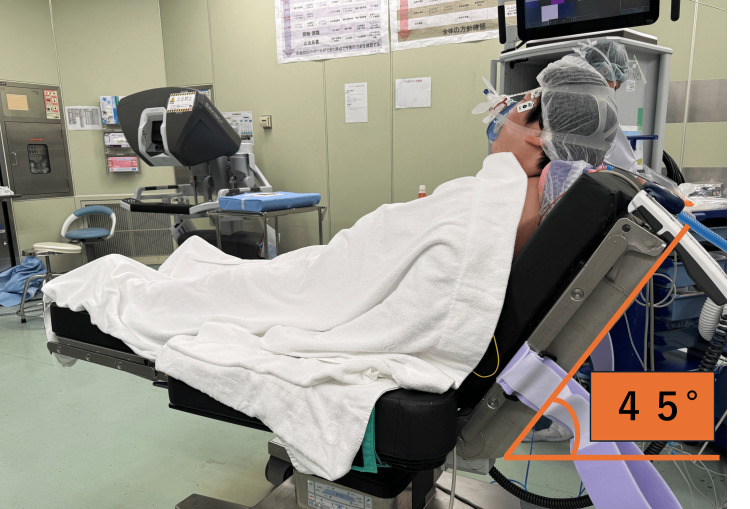
The patient was positioned in a 45° head-up position and received preoxygenation via face mask secured with an anesthesia mask head strap before induction of anesthesia.

As part of the preoperative anesthetic plan, sedation with remimazolam was initiated, and the patient was transitioned to the supine position while preserving spontaneous respiration in a semi-sitting position, followed by tracheal intubation. After arrival in the operating room, the patient remained calm in a 45° head-up (semi-sitting) position on the operating table (Figure 1); however, she was unable to tolerate the supine position because of severe low back pain. After the application of standard monitors, supplemental oxygen was administered via a facemask at 6 L/minute. Two minutes later, remimazolam infusion was initiated at 6 mg·kg⁻¹·hour⁻¹. At a total dose of 20 mg (three minutes after initiation), loss of response to verbal commands and tactile stimulation (shoulder tapping), absence of the eyelash reflex, and a bispectral index (BIS) value below 60 were confirmed. Spontaneous respiration was maintained, and the patient was then transferred to the supine position. Rocuronium (90 mg) and remifentanil (300 μg) were administered intravenously. Mask ventilation was easy. After confirming a train-of-four (TOF) count of zero, tracheal intubation was performed using a video laryngoscope, and mechanical ventilation was initiated with a fraction of inspired oxygen of 0.5 (Table 1). The Cormack-Lehane laryngoscopic view was grade III. No complications, including oxygen desaturation, were observed during induction or tracheal intubation. An ultrasound-guided posterior transversus abdominis plane block and a re-modified thoracoabdominal nerve block using a perichondrial approach (RM-TAPA) were performed for perioperative analgesia using 0.25% levobupivacaine. For the posterior transversus abdominis plane block, 10 mL was injected on each side, and for the RM-TAPA block, 20 mL was injected on each side, for a total volume of 60 mL. Anesthesia was maintained with remimazolam at 0.6 mg·kg⁻¹·hour⁻¹, remifentanil at 0.2 μg·kg⁻¹·minute⁻¹, and intermittent rocuronium administration. Intraoperatively, the BIS was maintained between 40 and 63, and the TOF count was maintained between 0 and 1. At wound closure, fentanyl 200 μg was administered intravenously, and acetaminophen 1,000 mg was initiated as a continuous intravenous infusion.

**Table 1:** Peri-induction vital signs and monitoring variables at key time points. Vital signs and monitoring values are shown at baseline before induction, at the start of induction, in the semi-sitting position, after repositioning to the supine position, and after tracheal intubation. MAP was calculated from the recorded systolic and diastolic blood pressures. A dash indicates that the value was not documented at the corresponding time point. TOF ratio values greater than 100% reflect normalization to baseline values. Abbreviations: BIS, bispectral index; DBP, diastolic blood pressure; HR, heart rate; MAP, mean arterial pressure; SBP, systolic blood pressure; SpO2, peripheral oxygen saturation; TOF, train-of-four

Variable	Before induction (0:00)	Start of induction (0:12)	Semi-sitting position (0:14)	Supine position (0:16)	After intubation (0:18)
HR, beats minute⁻¹	90	104	95	82	82
SBP, mmHg	181	200	165	158	147
DBP, mmHg	122	121	109	108	96
MAP, mmHg	142	147	128	125	113
SpO2, %	96	99	99	99	99
BIS	-	100	79	62	41
TOF ratio, %	-	104	102	104	-
TOF count	-	-	-	-	0

At the end of surgery, neuromuscular monitoring revealed a TOF count of 2/4. Sugammadex was administered at 2 mg·kg⁻¹, and recovery to a TOF ratio of 1.0 was confirmed. Flumazenil 0.25 mg was administered. After confirming adequate spontaneous ventilation, including a respiratory rate of 10 breaths/minute and a tidal volume of >6 mL/kg, the tracheal tube was removed. Following extubation, supplemental oxygen was provided via a face mask at 5 L/minute. The patient was placed in a 45° semi-sitting position during transfer to bed. No oxygen desaturation was observed, and lung auscultation revealed no abnormal findings or asymmetry. Upon emergence from anesthesia, the patient reported no incisional pain, nausea, vomiting, or back pain and left the operating room in a stable condition. The total anesthesia time was four hours and seven minutes, and the operation time was two hours and 49 minutes. Intraoperative blood loss, crystalloid infusion, and urine output were 22, 750, and 300 mL, respectively. Postoperatively, there was no worsening of neurological symptoms related to the prior lumbar fracture. The patient experienced no significant incisional pain, had an uneventful recovery, and was discharged on postoperative day four.

## Discussion

In the sitting position, gravitational forces promote blood pooling in the lower extremities and the splanchnic vascular bed, leading to reductions in venous return and cardiac output. The vasodilatory and myocardial depressant effects of anesthetic induction agents may further predispose patients to hypotension during induction, potentially increasing the risk of cerebral ischemia [1]. Therefore, careful hemodynamic management is required. Technical constraints associated with the sitting position may complicate mask ventilation and laryngoscopy. If ventilation or tracheal intubation becomes difficult, rapid repositioning may also be challenging, thereby increasing the risks associated with airway management [3]. Patients with morbid obesity are at an increased risk of comorbid conditions such as diabetes mellitus, hypertension, atrial fibrillation, and coronary artery disease. In addition, fat deposition in the facial, pharyngeal, and lingual regions is common, which may render mask ventilation and tracheal intubation more difficult than in normal-weight patients. Consequently, these patients are at a higher risk of perioperative respiratory and cardiovascular complications [4].

Remimazolam is an ultra-short-acting benzodiazepine characterized by relatively preserved spontaneous respiratory drive [5]. In addition, its suppressive effects on sympathetic activity and the baroreceptor reflex are less pronounced than those of other anesthetic agents, allowing mean arterial pressure to be better maintained during anesthesia [6]. Furthermore, the availability of a specific antagonist, flumazenil, enhances titratability and reversibility. In the present case, the purpose of using remimazolam was not to maintain spontaneous ventilation until tracheal intubation itself, but to preserve spontaneous respiration only until the patient could be safely transitioned from the semi-sitting position to the supine position. Once repositioning had been completed and mask ventilation was confirmed to be easy, we deliberately proceeded with rocuronium administration and tracheal intubation under full neuromuscular relaxation in order to achieve conventional optimal intubating conditions and maximize the likelihood of first-pass success. We considered this strategy particularly meaningful because the overall anesthetic plan remained controllable and potentially reversible: remimazolam could be antagonized with flumazenil, rocuronium with sugammadex, and remifentanil has a rapid offset profile. Thus, even if airway management had become unsafe, the patient could potentially have been returned to spontaneous respiration and awakening. Propofol is characterized by a rapid onset of action and reliable induction of hypnosis. However, its dose-dependent myocardial depressant and vasodilatory effects frequently result in hypotension through reductions in systemic vascular resistance and cardiac output. In the sitting or semi-sitting position, venous return is further decreased because of the gravitational redistribution of blood volume, leading to reduced preload and a relatively limited circulatory reserve. Excessive hypotension may therefore occur under these conditions [1]. Dexmedetomidine, another sedative agent with minimal respiratory depression and modest analgesic properties, was also considered during the transition from the sitting to the supine position. However, it may provide insufficient hypnosis for the induction of general anesthesia. In addition, its relatively slow pharmacokinetic profile may limit rapid titration, and the absence of a specific antagonist may reduce reversibility. Given the patient’s history of airway compromise under sedation, dexmedetomidine was deemed unsuitable in the present case.

In patients with obesity, the supine position increases intra-abdominal pressure, resulting in a marked reduction in functional residual capacity and shortened apnea tolerance [7]. In contrast, a head-up or sitting position reduces cephalad displacement of the diaphragm, thereby improving functional residual capacity and lung compliance, and enhancing the efficiency of preoxygenation. Previous studies have reported that a head-up position of approximately 25-30° is advantageous for optimizing oxygen reserves in patients with obesity [7]. In the present case, severe pain during positional changes precluded maintenance of the supine or even mild head-up position; therefore, anesthetic induction was performed in a 45° semi-sitting position, taking into account the patient’s tolerance. Although preoxygenation in a fully upright (90°) sitting position has been reported to provide the greatest prolongation of apnea tolerance [8], this position may be less practical for anesthetic induction and airway intervention in emergency situations. In shoulder surgery, it has been reported that induction of anesthesia in a reclining beach-chair position is associated with higher blood pressure and more stable hemodynamics than induction in a fully upright beach-chair position [1]. These findings suggest that a 45° semi-sitting position may preserve the physiological benefits of preoxygenation - namely improved oxygen reserve - without compromising the safety and feasibility of anesthetic induction and airway management.

Induction of anesthesia in the sitting position may facilitate maintenance of oxygenation, even in the presence of upper airway obstruction or hypoventilation, thereby providing a greater temporal and physiological safety margin for intervention.

If spontaneous respiration had been lost during induction in the sitting position, our plan was to attempt mask ventilation while maintaining the sitting or semi-sitting position as much as possible and to use airway adjuncts as needed to improve upper airway patency. If mask ventilation had been inadequate and upper airway obstruction had been considered the primary cause, the administration of a neuromuscular blocking agent would have been considered to improve ventilatory conditions. Tracheal intubation or insertion of a supraglottic airway device would also have been attempted. If these measures had failed, our fundamental strategy would have been to abort induction and allow the patient to regain consciousness and spontaneous respiration (waking the patient) [9,10]. For tracheal intubation in the semi-sitting position, a face-to-face technique or video laryngoscopy performed from behind the patient has been recommended [3]. However, given our limited experience with the face-to-face technique and reports of failed attempts using this approach [2], we considered video laryngoscopic intubation from behind the patient as an alternative strategy if needed. Fortunately, no significant ventilatory impairment occurred in this case. At the time of induction, we selected a reversible anesthetic agent to ensure that, even in the event of progression to a “cannot intubate, cannot oxygenate” (CICO) situation, rapid awakening and restoration of spontaneous respiration would be possible. This approach allowed us to maintain an adequate safety margin even under the assumption of a worst-case scenario.

## Conclusions

In this patient with obesity who was unable to tolerate the supine position because of severe pain, induction of anesthesia was safely accomplished using remimazolam while transitioning from the sitting to the supine position. When performing anesthetic induction in a nonstandard position, careful preoperative planning - including appropriate selection of sedative agents and preparation for contingency airway management - is essential.
